# Amyloid PET quantification using low-dose CT-guided anatomic standardization

**DOI:** 10.1186/s13550-021-00867-7

**Published:** 2021-12-14

**Authors:** Hiroshi Matsuda, Tensho Yamao, Mitsuru Shakado, Yoko Shigemoto, Kyoji Okita, Noriko Sato

**Affiliations:** 1 Department of Biofunctional Imaging, Fukushima Medical University, 1 Hikariga-oka, Fukushima City, Fukushima 960-1295 Japan; 2 Drug Discovery and Cyclotron Research Center, Southern Tohoku Research Institute for Neuroscience, 7-61-2 Yatsuyamada, Koriyama, Fukushima 963-8052 Japan; 3 Department of Radiology, National Center of Neurology and Psychiatry, 4-1-1 Ogawahigashi, Kodaira, Tokyo 187-8551 Japan; 4 Department of Radiological Sciences, School of Health Sciences, Fukushima Medical University, 10-6, Sakae, Fukushima 960-8516 Japan; 5 Integrative Brain Imaging Center, National Center of Neurology and Psychiatry, 4-1-1 Ogawahigashi, Kodaira, Tokyo 187-8551 Japan; 6 Department of Biofunctional Imaging, Fukushima Medical University, 6F(621), Shin-Otemachi Building, 2-2-1, Otemachi, Chiyoda-ku, Tokyo 100-0004 Japan

**Keywords:** Alzheimer’s disease, Amyloid imaging, Positron emission tomography, Magnetic resonance imaging, Computed tomography, Centiloid scale

## Abstract

**Background:**

Centiloid (CL) scaling has become a standardized quantitative measure in amyloid PET because it facilitates the direct comparison of results across institutions, even when different analytical methods or tracers are used. Standard volumes of interest must be used to calculate the CL scale after the anatomic standardization of amyloid PET images using coregistered MRI; if the MRI is unavailable, the CL scale cannot be accurately calculated. This study sought to determine the substitutability of low-dose CT, which is used to correct PET attenuation in PET/CT equipment, by evaluating the measurement accuracy when low-dose CT is used as an alternative to MRI in the calculation of the CL scale. Amyloid PET images obtained using ^18^F-flutemetamol from 24 patients with possible or probable Alzheimer’s disease were processed to calculate the CL scale using 3D T1-weighted MRI and low-dose CT of PET/CT. CL_MRI_ and CL_CT_ were, respectively, defined as the use of MRI and CT for anatomic standardization and compared. Regional differences in the CT-based and MRI-based standardized anatomic images were also investigated. Trial registration: Japan Registry of Clinical Trials, jRCTs031180321 (registered 18 March 2019, https://jrct.niph.go.jp/latest-detail/jRCTs031180321).

**Results:**

A Bland–Altman plot showed that CL_CT_ was slightly but significantly underestimated (mean ± standard deviation, − 1.7 ± 2.4; *p* < 0.002) compared with CL_MRI_. The 95% limits of agreement ranged from − 2.8 to − 0.7. Pearson correlation analysis showed a highly significant correlation of *r* = 0.998 between CL_CT_ and CL_MRI_ (*p* < 0.001). The linear regression equation was CL_MRI_ = 1.027 × CL_CT_ + 0.762. In a Bland–Altman plot, Spearman correlation analysis did not identify a significant association between the difference in CL_MRI_ versus CL_CT_ and CL load (*ρ* =  − 0.389, *p* = 0.060). This slight underestimation of CL_CT_ may derive from slightly higher uptake when the cerebellum is used as a reference area in CT-based anatomically standardized PET images versus MRI-based images.

**Conclusions:**

Low-dose CT of PET/CT can substitute for MRI in the anatomic standardization used to calculate the CL scale from amyloid PET, although a slight underestimation occurs.

## Background

In clinical practice for dementia, amyloid PET increases the certainty of Alzheimer's disease (AD) and non-AD diagnosis [1]. In many settings, the binary classification of positive and negative amyloid PET findings is based on visual interpretation. Equivocal findings are thus inevitable and lead to interrater variability in visual interpretation [2] because raters have their own experience and potential internal criteria. In addition, in our previous multicenter study using ^18^F-flutemetamol [3], disagreement between two raters was observed in 9% of cases. Equivocal findings should be avoided when determining the indication for the disease-modifying drugs currently in development. Accordingly, quantitative analysis has been proposed as an adjunct to visual interpretation [4].

The quantitative analysis of amyloid PET has widely applied the standardized uptake value ratio (SUVR). However, SUVR values vary not only according to the target and reference regions used, but also according to the particular amyloid tracer used. This variability can be resolved through a Centiloid (CL) scaling process that standardizes the quantitative amyloid imaging measures by standardizing the outcome of each analytical method or PET ligand to a scale from 0 to 100 [5]. The CL scaling method can facilitate the direct comparison of results across institutions, even when different analytical methods or tracers are used, and may enable cutoffs for amyloid positivity to be clearly defined. A positive relationship of the CL scale cutoff with pathological findings has been found [6–9], and it is expected that the clinical value of the CL scale will continue to increase. When determining the CL scale, it is necessary to follow the method advocated by the Global Alzheimer’s Association Interactive Network (GAAIN, http://www.gaain.org/centiloid-project). Because highly accurate anatomic standardization to standard Montreal Neurological Institute (MNI) space is required to use standard volumes of interest (VOIs) supplied from GAAIN to calculate the CL scale converted from SUVR, anatomic standardization of amyloid PET images should be performed using coregistered MRI obtained around the same period. For this MRI, the use of a three-dimensional (3D) T1-weighted image that covers the whole brain is recommended. However, if a 3D T1-weighted image is not available, the CL scale cannot be accurately calculated. As an alternative, Pressoto et al. [10] reported that anatomic standardization of amyloid PET can be performed with low-dose CT, which is used to correct PET attenuation in PET/CT equipment. According to their report, the difference between the SUVR values measured by anatomic standardization using MRI and low-dose CT is only 0.01 ± 0.03 (mean ± standard deviation) and is thus negligible. The purpose of the present study was to establish the substitutability of low-dose CT of PET/CT proposed by Pressoto et al. by verifying the measurement accuracy at the global and regional levels when low-dose CT is used as an alternative to MRI in the calculation of the SUVR and CL scales in amyloid PET.

## Materials and methods

### Participants

The study participants comprised 24 patients (15 men and 9 women; age range 48–90 years) enrolled in a previous multicenter study [3]. They were recruited from an outpatient memory clinic of the National Center of Neurology and Psychiatry, Japan. The participants had a Mini-Mental State Examination score of 19.7 ± 4.6 and a global Clinical Dementia Rating of 0.8 ± 0.4. According to National Institute on Aging and the Alzheimer’s Association criteria [11], 10 and 14 patients were diagnosed as having possible and probable AD, respectively.

### Image acquisition

#### ^18^F-flutemetamol PET/CT

Each subject received an intravenous injection of 215 ± 33 MBq of ^18^F-flutemetamol (Vizamyl, Nihon Medi-Physics). All PET acquisitions were performed using a hybrid PET/CT Biograph 16 True-point scanner (Siemens Healthineers, Erlangen, Germany). After positioning, a low-dose CT scan (kVp, 130 keV; current, 40 mA; rotation time, 1.0 s; table feed per rotation, 7.2 mm; spiral pitch factor, 0.75) was acquired to be used for the attenuation correction of the PET data. Images were reconstructed using the “H10s very smooth” kernel, a 30.0-cm reconstruction field of view, and a 2.0-mm slice interval for a resulting voxel size of 0.59 × 0.59 × 2.0 mm^3^. A 3D-PET acquisition (list mode) was started 61.2 ± 0.8 min after the injection of the tracer and lasted for 20 min. Image reconstruction was performed using a 3D ordered subsets expectation maximization algorithm with the following parameters: image matrix, 168; field of view, 300 mm; subsets, 21; iterations, 4; post-filter (Gaussian), 4-mm FWHM; attenuation correction, CT-based. The resulting voxel size was 2.02 × 2.06 × 2.03 mm^3^. This low-dose CT protocol delivers a head radiation dose of 0.4 mSv.

#### MRI

The MRI for all patients was performed on an Achieva 3.0-T MR scanner (Philips Medical Systems, Best, The Netherlands) equipped with a 32-channel coil within 42 ± 21 days before the amyloid PET. A volumetric turbo field echo T1-weighted structural sequence (300 sagittal slices; TR, 7.0 ms; TE, 3.4 ms; field of view, 260 × 240 mm; voxel size, 0.7 × 0.7 × 0.6 mm^3^; flip angle, 10°) was acquired for each subject.

### Quantitative analysis

**Fig. 1:**
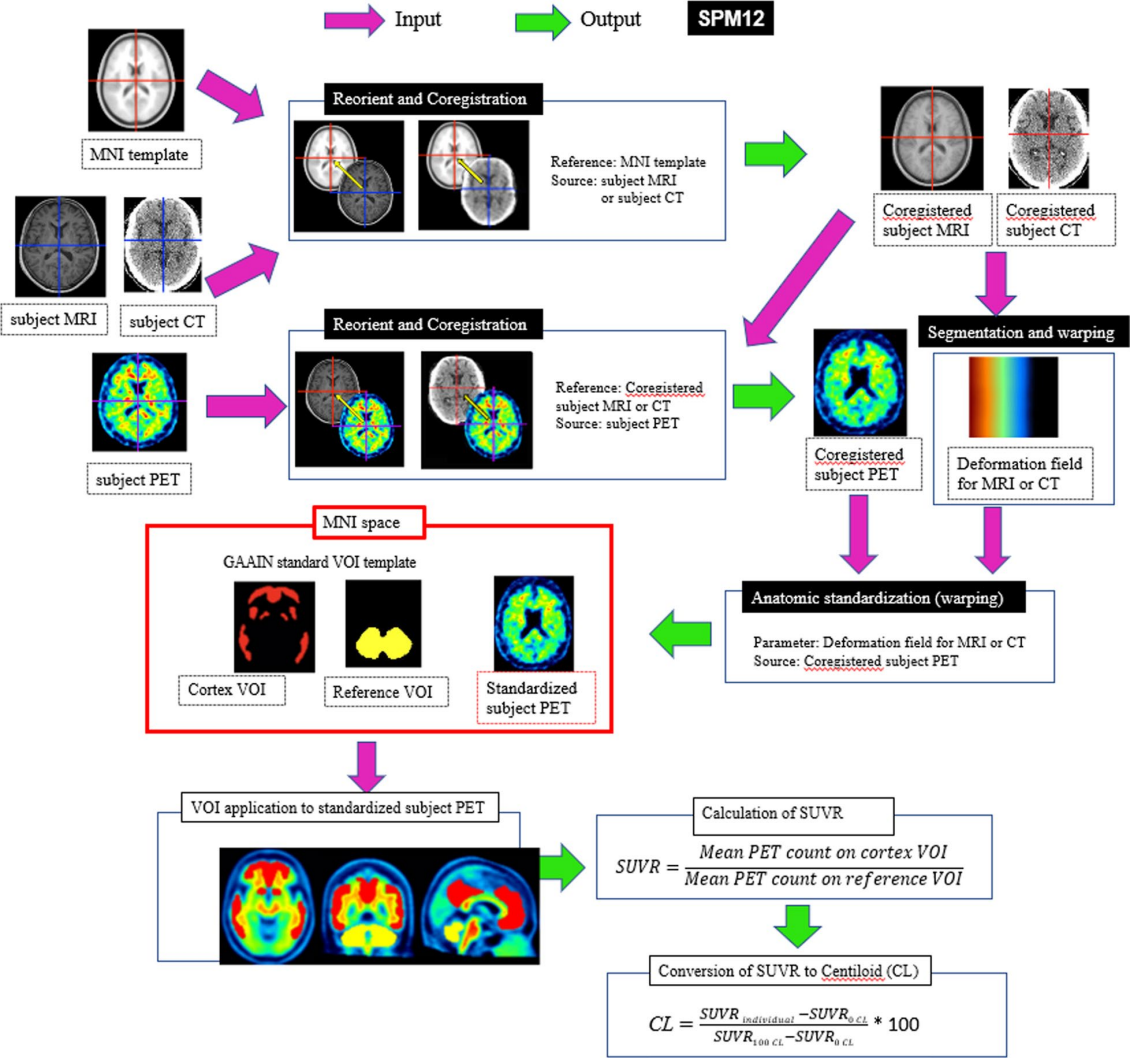
Processing pipeline for quantitative measurements of ^18^F-flutemetamol accumulation in the cerebral cortex. The subject MRI or CT was oriented and coregistered to the MNI template. The subject PET was oriented and coregistered to the coregistered subject MRI or subject CT. Then, the coregistered subject MRI or subject CT was warped into MNI space using unified segmentation in SPM12. The parameters of the deformation field in this warping were applied to the coregistered subject PET for anatomical standardization into MNI space. SUVR was calculated from the ^18^F-flutemetamol PET counts in the cerebral cortical areas (Cortex VOI, CTX VOI) and in the whole cerebellum as a reference area (Reference VOI, WhlCbl VOI) using the GAAIN standard VOI template. Then, SUVR was converted to CL using a direct conversion equation. Processing with a black background was performed using Statistical Parametric Mapping (SPM) 12

Figure 1 shows the processing pipeline applied to quantitative analysis using SUVR and the 100-point CL scale [5]. This MRI-based pipeline has already been validated [3] using a GAAIN dataset of ^11^C-PiB PET images for 34 young control individuals and 45 typical AD patients downloaded from the GAAIN website. In the present study, low-dose CT was also used instead of MRI for anatomic standardization. The CL scale assigns an average value of 0 to high-certainty amyloid-negative subjects and an average of 100 to typical AD patients. First, in this pipeline, the subject MRI or CT was oriented and coregistered to the MNI template (avg152T1.nii). The subject PET was then oriented and coregistered to the coregistered subject MRI or CT. Then, the coregistered subject MRI or CT was warped into MNI space using unified segmentation [12]. These translations were performed using the Statistical Parametric Mapping (SPM) 12 software (https://www.fil.ion.ucl.ac.uk/spm). The parameters of the deformation field in this warping were applied to the coregistered subject PET for anatomic standardization into MNI space. Using the standard VOI in GAAIN, SUVR was calculated from ^18^F-flutemetamol PET counts in the global cortical target area (GAAIN, CTX VOI) and in the whole cerebellum (GAAIN, WhlCbl VOI) as the reference area. Then, a direct conversion equation (CL = 121.42 × SUVR − 121.16) was applied to convert SUVR to the CL value, as described previously [13].

In addition, PET-only methods using a mean atlas and an adaptive atlas as a template for anatomic standardization in SPM12 were compared against MRI-based quantification in the same manner as previously reported [14]. These PET templates were generated using the present MRI-based anatomical standardization method from datasets collected from the publicly available GAAIN repository. Positive and negative ^18^F-flutematamol templates as an adaptive atlas were generated by averaging images from 28 AD patients with a CL scale of 92.0 ± 21.6 and 23 young healthy controls with a CL scale of − 1.0 ± 3.5, respectively. A mean atlas was generated by averaging the images of these AD patients and young healthy controls with a CL scale of 50.1 ± 49.4. The selection of the adaptive atlas for the positive or negative template was based on the amyloid-positive cutoff CL level of 16 in a previous report [3].

We defined SUVR_MRI_, SUVR_CT_, SUVR_mPET_, and SUVR_aPET_, as well as CL_MRI_, CL_CT_, CL_mPET_, and CL_aPET_, based on the use of MRI, CT, mean PET, and adaptive PET for anatomic standardization, respectively.

### Endpoints

The endpoint of this study was the measurement accuracy of SUVR_CT_ and CL_CT_ when SUVR_MRI_ and CL_MRI_ were, respectively, regarded as the gold standard.

### Statistical analysis

Concordances between SUVR_MRI_ and SUVR_CT_, SUVR_MRI_ and SUVR_mPET_, and SUVR_MRI_ and SUVR_aPET_ and between CL_MRI_ and CL_CT_, CL_MRI_ and CL_mPET_, and CL_MRI_ and CL_aPET_ were assessed using Bland–Altman plots and Pearson correlation estimates. In the Bland–Altman plot of SUVR and CL, we used Spearman correlation analysis to examine whether there were associations between the difference in SUVR_MRI_ versus SUVR_CT_ and SUVR load and between the difference in CL_MRI_ versus CL_CT_ and CL load. Spearman correlation analysis was also performed for SUVR and CL obtained from PET-only methods. SUVR and CL and their standard deviations were computed with mean absolute differences and limits of agreement. These statistical tests were performed using JMP ver. 16 (SAS Institute). In addition, to investigate regional differences in the CT-based and MRI-based standardized amyloid PET images, a paired t-test was applied to these images on a voxel basis after smoothing with an 8-mm FWHM Gaussian kernel using SPM12. Results were considered significant at *p* < 0.001 with an extent threshold of 300 voxels without multiple comparisons.

## Results

**Fig. 2:**
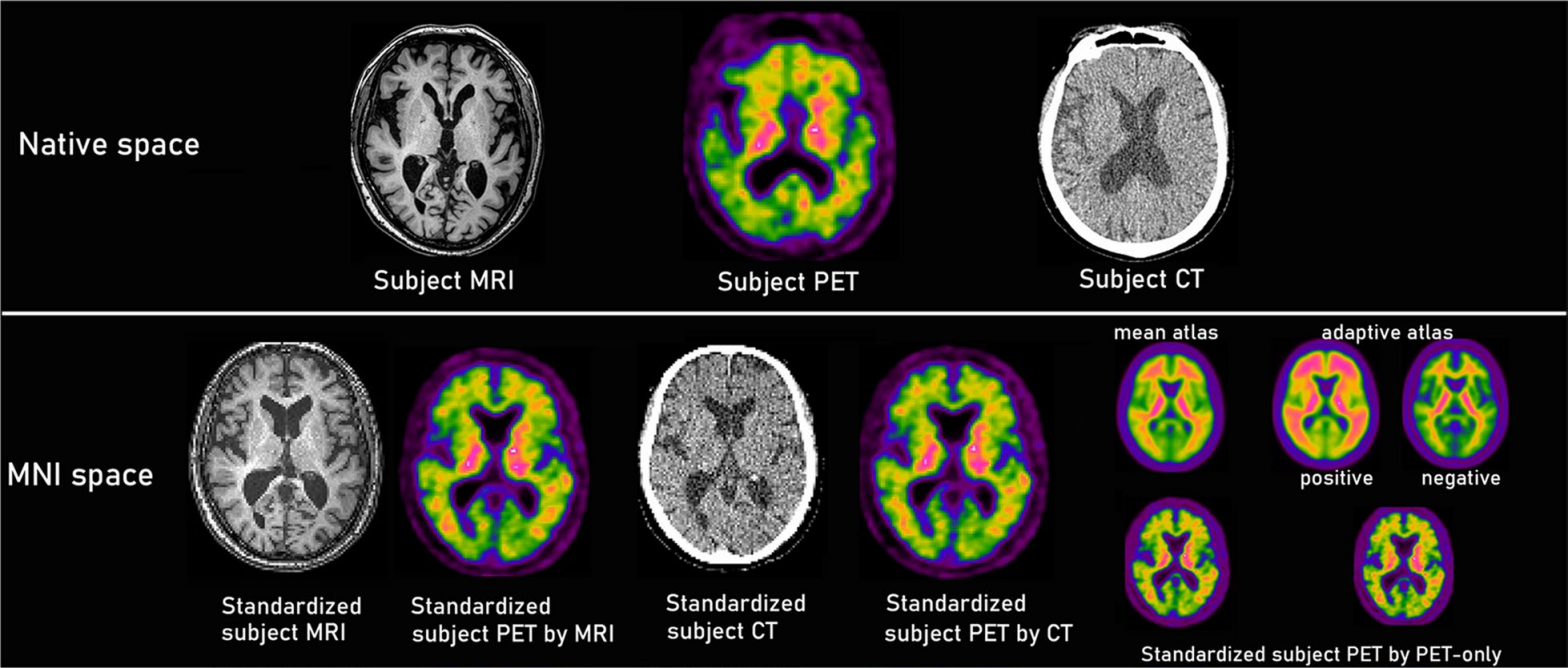
Coregistered (upper row, native space) and anatomically standardized (lower row, MNI space) MRI, PET, and CT images. Almost identical PET images were obtained after anatomic standardization between the MRI-based and CT-based approaches. The PET-only approach involved anatomic standardization using a mean atlas for a single template or an adaptive atlas for a positive or negative template

**Fig. 3:**
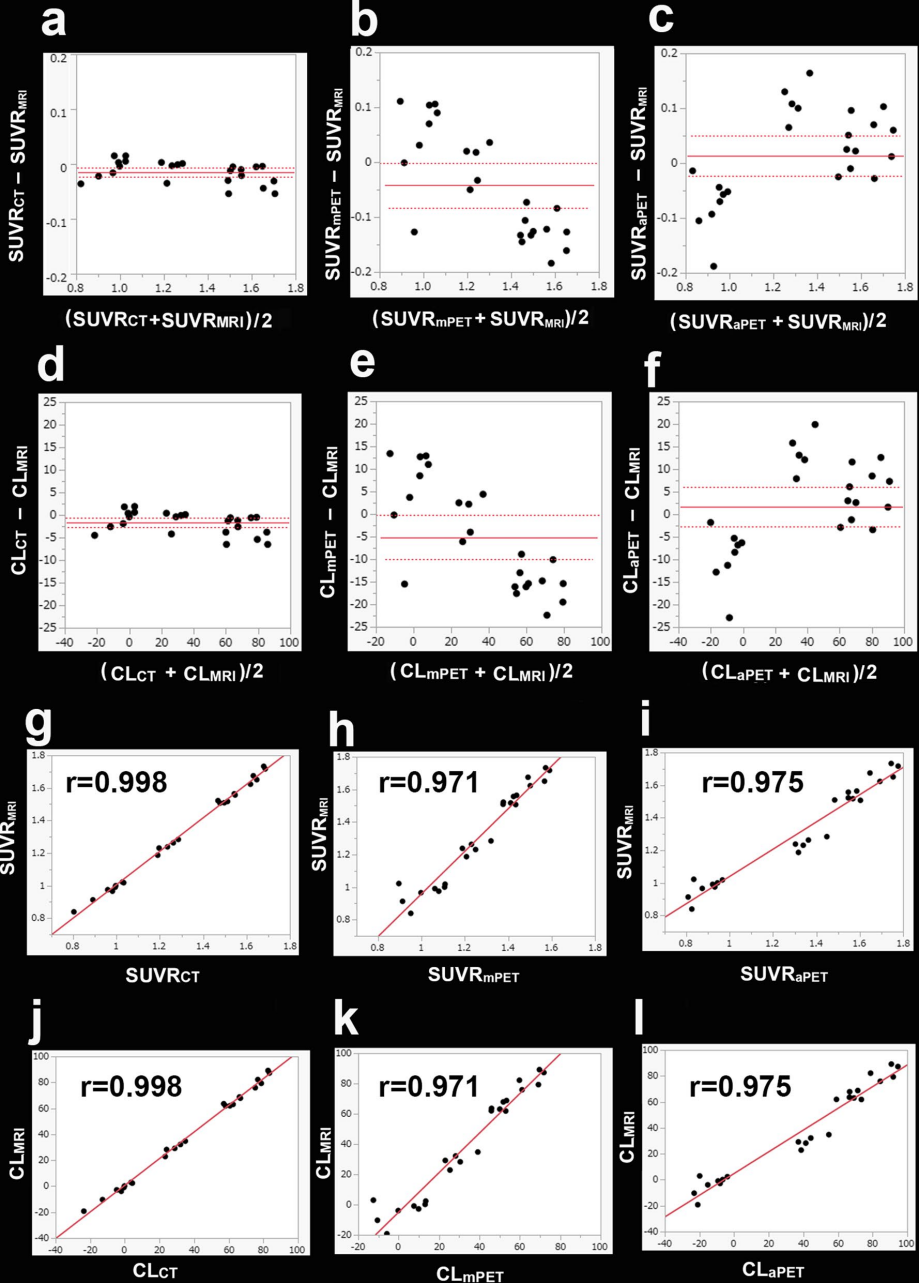
Comparison of the SUVR and CL values obtained from the MRI-based, CT-based, and PET-only approaches. Bland–Altman plots **a**, **d** showed slight but significant underestimation of SUVR_CT_ and CL_CT_ compared with SUVR_MRI_ and CL_MRI_, respectively (*p* < 0.002). Spearman correlation analysis did not show a significant association between the difference in SUVR_MRI_ versus SUVR_CT_ and SUVR load (*ρ* =  − 0.379, *p* = 0.051) and between the difference in CL_MRI_ versus CL_CT_ and CL load (*ρ* =  − 0.389, *p* = 0.060). Pearson correlation analysis **g**, **j** showed highly significant correlations of *r* = 0.998 between SUVR_CT_ and SUVR_MRI_ and between CL_CT_ and CL_MRI_ (*p* < 0.001). Bland–Altman plots **b**, **e** showed significant underestimation of SUVR_mPET_ and CL_mPET_ compared with SUVR_MRI_ and CL_MRI_, respectively (*p* < 0.05). Spearman correlation analysis showed a significant association between the difference in SUVR_MRI_ versus SUVR_mPET_ and SUVR load (*ρ* =  − 0.713, *p* < 0.001) and between the difference in CL_MRI_ versus CL_mPET_ and CL load (*ρ* =  − 0.702, *p* < 0.001). Pearson correlation analysis **h**, **k** showed highly significant correlations of *r* = 0.971 between SUVR_mPET_ and SUVR_MRI_ and between CL_mPET_ and CL_MRI_ (*p* < 0.001). Bland–Altman plots **c**, **f** showed a tendency for overestimation of SUVR_aPET_ and CL_aPET_ compared with SUVR_MRI_ and CL_MRI_, respectively (*p* > 0.2). Spearman correlation analysis showed a significant association between the difference in SUVR_MRI_ versus SUVR_aPET_ and SUVR load (*ρ* = 0.515, *p* < 0.001) and between the difference in CL_MRI_ versus CL_mPET_ difference and CL load (*ρ* = 0.515, *p* < 0.001). Pearson correlation analysis **i**, **l** showed significant correlations of *r* = 0.975 between SUVR_aPET_ and SUVR_MRI_ and between CL_aPET_ and CL_MRI_ (*p* < 0.001)

Figure 2 shows the standardized MRI, CT, and amyloid PET images in MNI space in a subject, along with the three corresponding images in native space. When compared with SUVR_MRI_ and CL_MRI_ using a Bland–Altman plot (Fig. 3a, d), SUVR_CT_ and CL_CT_ were slightly but significantly underestimated (− 0.01 ± 0.02 and − 1.7 ± 2.4, respectively; *p* < 0.002). The 95% limits of agreement ranged from − 0.02 to − 0.01 for SUVR and from − 2.8 to − 0.7 for CL. Probable AD patients showed greater underestimation of SUVR_CT_ and CL_CT_ (− 0.02 ± 0.02 and − 2.4 ± 2.7, respectively; *p* < 0.003) compared with underestimation of SUVR_CT_ and CL_CT_ in possible AD patients (− 0.01 ± 0.01 and − 0.8 ± 1.6, respectively; *p* = 0.08). Pearson correlation analysis (Fig. 3g, j) showed a highly significant correlation of *r* = 0.998 between SUVR_CT_ and SUVR_MRI_ (*p* < 0.001). The linear regression equation was SUVR_MRI_ = 1.027 × SUVR_CT_ − 0.020. CL_CT_ also showed a highly significant correlation of r = 0.998 with CL_MRI_ (*p* < 0.001). The linear regression equation was CL_MRI_ = 1.027 × CL_CT_ + 0.762.

Compared with SUVR_MRI_ and CL_MRI_ using a Bland–Altman plot (Fig. 3b, e), SUVR_mPET_ and CL_mPET_ were significantly underestimated (− 0.04 ± 0.10 and − 5.1 ± 11.7, respectively; *p* < 0.05). The 95% limits of agreement ranged from − 0.08 to 0.0 for SUVR and from − 9.8 to − 0.4 for CL. Pearson correlation analysis (Fig. 3h, k) showed a significant correlation of *r* = 0.971 between SUVR_mPET_ and SUVR_MRI_ and between CL_mPET_ and CL_MRI_ (*p* < 0.001).

Compared with SUVR_MRI_ and CL_MRI_ using a Bland–Altman plot (Fig. 3c, f), SUVR_aPET_ and CL_aPET_ showed a tendency for overestimation (0.01 ± 0.09 and 1.6 ± 10.3, respectively; *p* > 0.2). The 95% limits of agreement ranged from − 0.02 to + 0.05 for SUVR and from − 2.5 to + 5.8 for CL. Pearson correlation analysis (Fig. 3i, l) showed a significant correlation of *r* = 0.975 between SUVR_aPET_ and SUVR_MRI_ and between CL_aPET_ and CL_MRI_ (*p* < 0.001).

In a Bland–Altman plot, Spearman correlation analysis failed to identify a significant association between the difference in SUVR_MRI_ versus SUVR_CT_ and SUVR load (*ρ* =  − 0.379, *p* = 0.051) or between the difference in CL_MRI_ versus CL_CT_ and CL load (*ρ* =  − 0.389, *p* = 0.060). On the other hand, Spearman correlation analysis revealed a significant association between the difference in SUVR_MRI_ versus SUVR_mPET_ and SUVR load (*ρ* =  − 0.713, *p* < 0.001), between the difference in CL_MRI_ versus CL_mPET_ and CL load (*ρ* =  − 0.702, *p* < 0.001), between the difference in SUVR_MRI_ versus SUVR_aPET_ and SUVR load (*ρ* = 0.515, *p* < 0.001), and between the difference in CL_MRI_ versus CL_aPET_ and CL load (*ρ* = 0.515, *p* < 0.001).

**Fig. 4:**
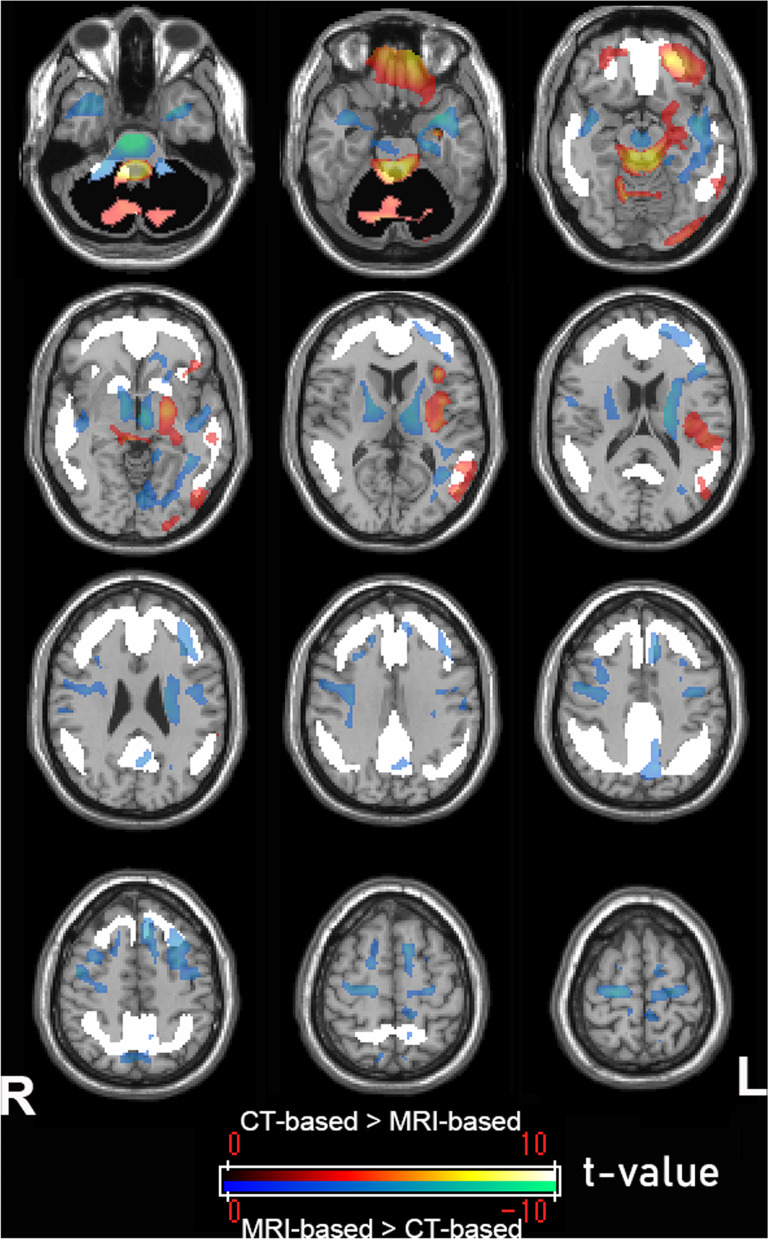
Direct comparison of anatomically standardized amyloid PET images using CT and MRI. SPM analysis showed significantly (*p* < 0.001) higher and lower uptake of CT-based standardized PET images than MRI-based standardized PET images presented in a warm color scale (*t* value, from 0 to 10) and a cool color scale (*t* value, from 0 to − 10), respectively. The largest differences in the accumulation were visible in the brain stem. Higher uptake of CT-based standardized PET images was observed within the whole cerebellar VOI as a reference area (solid black area). In the supratentorial area, most of the significant differences in uptake were found outside the cortical target VOI (solid white area)

Paired *t* tests performed using SPM12 (Fig. 4; Table 1) found that the brainstem exhibited the biggest differences in uptake between the CT-based and MRI-based standardized PET images. Higher uptake of CT-based standardized PET images than MRI-based PET images was observed within the whole cerebellar VOI. In the supratentorial area, most of the statistically significant differences in uptake between the CT-based and MRI-based standardized PET images were found outside of the global cortical target region VOI.

**Table 1:** Significant voxel-wise differences in CT-based and MRI-based standardized PET images

		Cluster size (no. of voxels)	*T* value (peak voxel)	MNI coordinates (*x*, *y*, *z*)	Location of peak voxels
MRI-based > CT-based		2408	11.94	2, − 18, − 38	Ventral brain stem
		2330	7.9	− 32, 6, − 24	Left temporal pole
		2335	7.08	0, − 12, − 8	Venral diencephalon
		1025	6.57	20, − 22, 66	Right precentral gyrus
		527	6.53	− 8, 24, 48	Left cerebral white matter
		621	6.27	− 44, 24, 16	Left cerebral white matter
		940	6.2	42, 6, − 32	Right cerebral white matter
CT-based > MRI-based		7097	12.87	0, − 48, − 54	Dorsal brain stem
		850	6.44	12, − 62, − 14	Right cerebellum exterior
		880	4.95	− 56, − 40, 2	Left middle temporal gyrus
		430	4.68	− 28, − 78, − 46	Left cerebellum exterior

## Discussion

This study confirmed that low-dose CT of PET/CT can substitute for MRI in the anatomic standardization performed to calculate the CL scale from amyloid PET. The average difference between SUVR_CT_ and SUVR_MRI_ was only 0.01, which was the same as in a previous study [10]. The difference in the CL scale between CL_CT_ and CL_MRI_ was also only 1.7 on average. CT acquired at exactly the same time as amyloid PET enables accurate coregistration of CT and PET. When MRI is unavailable, simultaneously obtained low-dose CT of PET/CT can provide accuracy nearly equal to that of MRI for CL scale calculation. This simultaneity can avoid the misregistration of MRI and PET when there is a substantial delay between modalities during which brain atrophy progresses in AD patients. On the other hand, the concordance between the SUVR and CL scales determined by PET-only methods and those determined by an MRI-based method was much lower than that between the SUVR and CL scales determined by a CT-based method and those determined by an MRI-based method.

Several studies have reported anatomic standardization using the amyloid PET template alone without MRI. However, the single-atlas PET template-based method provides a less accurate definition of cortical gray matter regions compared with the MRI-based method [15]. This inaccuracy may result from intensity-based standardization using a PET template. The high white matter and low gray matter uptake in amyloid-negative individuals tends to slightly shift the target’s white matter toward the single-atlas gray matter, leading to increased sampling of the white matter and an overestimation of the neocortical uptake. Mean cortical SUVRs in healthy controls were 1.11 and 1.27 on average using the single-atlas PET template and MRI-based method, respectively [15]. Similarly, the low white matter and high gray matter uptake in amyloid-positive individuals tends to slightly shift the target’s gray matter toward the single-atlas white matter, leading to decreased sampling of the gray matter and an underestimation of the neocortical uptake. Mean cortical SUVRs in AD patients were 1.76 and 1.74 on average using the single-atlas PET template and MRI-based method, respectively [15]. Consequently, the single-atlas PET template-based method gives a lower discrimination performance between cognitively normal individuals and AD patients compared with the MRI-based method [16]. The present PET-alone study using a mean atlas also revealed much greater underestimation of the SUVR and CL scales in comparison with the CT-based method. Moreover, this difference became greater as the SUVR or CL scale increased.

To overcome the drawback of the single-atlas PET template-based method, the application of a multi-atlas PET template to the anatomic standardization has been proposed [14, 17]. Computation of similarities between the anatomically standardized image of a patient and the multi-atlas templates automatically chooses the appropriate PET template. This multi-atlas approach reduces the overall error from 5.6% using a single-atlas to 2.7% in neocortical SUVR estimation compared with the MRI-based method [14]. Although this multi-atlas approach may improve the accuracy of anatomic standardization, it might not be able to cope with amyloid PET images with asymmetrical accumulation between the left and right hemispheres. This asymmetry causes interhemispheric differences in the accuracy of the registration to the PET template. In the present PET-alone method using an atlas adapted to a positive or negative template, there was a marked tendency for overestimation on the higher SUVR and CL scales. From these considerations, structural images may be necessary for precise anatomic standardization in amyloid PET.

CT-based standardization showed slightly but significantly lower SUVR and CL scales compared with MRI-based standardization. Direct comparison indicated that CT evaluation exhibits increased uptake in bilateral cerebellar hemispheres compared with MRI. This slightly increased uptake of the cerebellar hemisphere in a reference area may lead to a slight decrease in SUVR and CL in a target area of the cerebral cortex in CT-based standardization. This may be due to the lower accuracy of anatomic standardization by low-dose CT in the posterior cranial fossa compared with MRI.

There are some limitations in this study. First, we did not study the influence of the CT image quality on the anatomic standardization. Future studies will need to assess both the lower dose limit at which the algorithm still performs correctly with reduced current and whether the use of diagnostic-quality CT provides improvements. Second, the SPM results showed a remarkable difference in brain stem uptake between MRI-based and CT-based standardized images. Accordingly, the reference region should not be located in the brain stem, including the pons, in a CT-based method. Third, we applied this low-dose CT-guided method to only possible and probable AD patients with mild or moderate cognitive impairment. However, the greater difference in the SUVR and CL scales between the MRI-based and CT-based methods in probable AD patients than in AD patients suggests that future studies are needed to validate this method for the full spectrum of AD with varying levels of atrophy. Fourth, this approach should be further validated for the measurement of CL scales using other amyloid PET tracers, such as ^11^C-PiB, ^18^F-florbetapir, ^18^F-florbetaben, and ^18^F-NAV4694.

## Conclusions

This study proposed the use of low-dose CT of PET/CT for calculating the CL scale from amyloid PET. CL scales based on low-dose CT show a highly significant positive correlation with those based on MRI, regardless of the degree of amyloid accumulation in possible and probable AD patients with mild or moderate cognitive impairment. A key advantage of the use of low-dose CT is the simultaneous acquisition of PET and CT. This method would be applicable to subjects who are unable to undergo MRI.

## Data Availability

The datasets used and/or analyzed during the current study are available from the corresponding author on reasonable request.
